# Septochoanal Polyp on the Concave Side of the Deviated Nasal Septum

**DOI:** 10.7759/cureus.38427

**Published:** 2023-05-02

**Authors:** Yuki Numano, Kazuhiro Nomura, Mika Watanabe, Tomotaka Hemmi, Mitsuru Sugawara

**Affiliations:** 1 Otolaryngology, Tohoku University Hospital, Sendai, JPN; 2 Otolaryngology, Tohoku Kosai Hospital, Sendai, JPN; 3 Pathology, Tohoku Kosai Hospital, Sendai, JPN

**Keywords:** pathology, nasal septum deviation, allergy, septochoanal polyp, choanal polyp

## Abstract

The septochoanal polyp is one of the choanal polyps derived from the nasal septum. They rarely occur, with only a few cases reported in the English literature. The etiology is still uncertain though it is thought to be associated with inflammation. Pathological findings generally show chronic inflammatory polyps and should be differentiated from other tumors. We report a case of a 32-year-old man diagnosed with a septochoanal polyp on the concave side of the deviated nasal septum. Previous reports have not mentioned the relationship between septochoanal polyp and the direction of septal deviation. This case is a practical example when considering the potential causes of the septochoanal polyp.

## Introduction

Choanal polyps are one form of nasal polyps that extend toward the choana [1]. They are common unilateral benign masses usually originating from paranasal sinuses, such as maxillary, ethmoid, and sphenoid sinuses [1]. On the other hand, the septochoanal polyp is derived from the nasal septum. Choanal polyp arising from the nasal septum is extremely rare and termed septochoanal polyp, with only a few cases reported in English. The pathogenesis is still uncertain [2]. The symptoms are nasal obstruction, anosmia, rhinorrhea, and snoring [3]. The major differential diagnoses to consider with septochoanal polyp are epithelial adenomatoid hamartoma (REAH), chordoma, angiofibroma, paraganglioma, teratoma, and papilloma. Pathological examination reveals chronic inflammatory polyp, which is essential to differentiate from other tumors [4]. The standard treatment is surgical excision. Removal of polyps, including the small part of normal mucosa surrounding the origin, is essential to impede the recurrence [5].

We herein report a 32-year-old man diagnosed with a septochoanal polyp on the concave side of the deviated nasal septum. This case is a practical example of pondering the pathogenesis of septochoanal polyp and their characteristics compared to the other tumors.

## Case presentation

**Figure 1:**
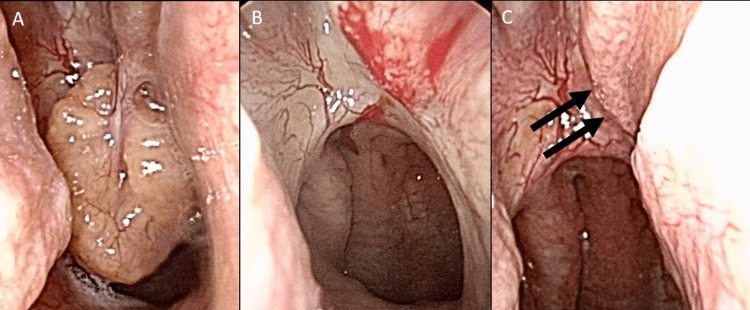
Endoscopic findings of the right nasal cavity pre- and post-treatment A. A white-yellow mass originates from the posterior nasal septum extending into the nasopharynx. B. After the mass was totally removed, together with the normal mucosa of the right septum. C. Three weeks after the surgery. The lesion at the right nasal septum is replaced with the healthy mucosa

A 32-year-old man presented to our hospital after a polyp in the right nasal cavity was found at the otolaryngology clinic near his home. He had not complained of rhinorrhea or nasal pain but right nasal obstruction for three months. He had no history of allergic disease. We performed an endoscopic examination and found a white-yellow mass arising from the posterior part of the nasal septum and extending into the nasopharynx (Figure 1A).

**Figure 2:**
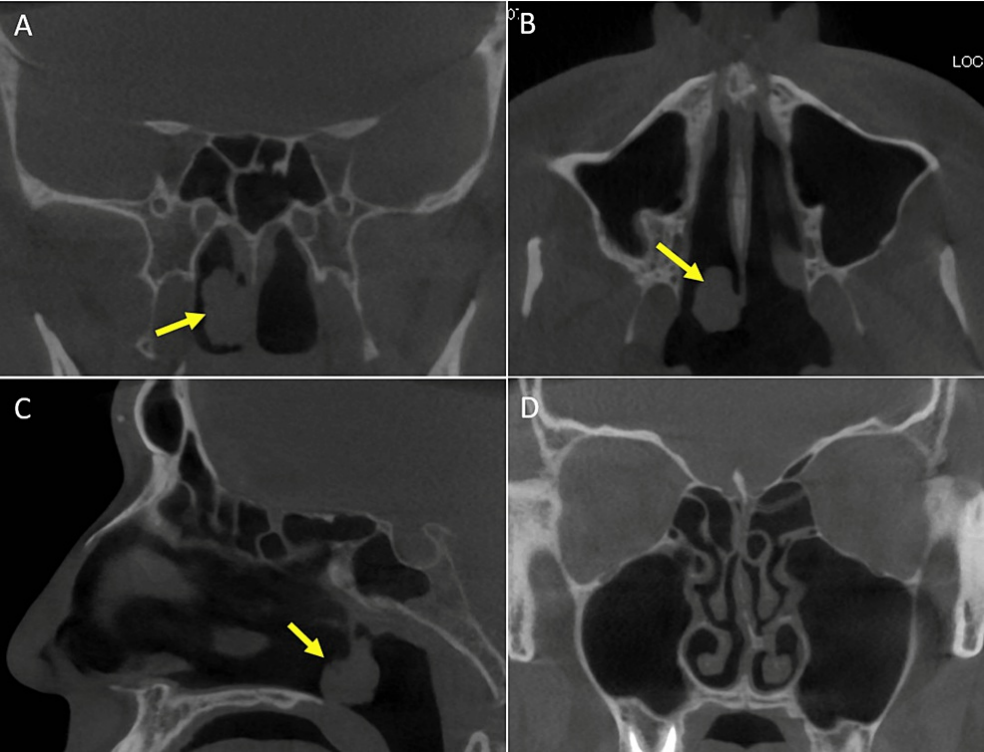
Computed tomography findings. A coronal image B. axial image C. sagittal image (A) coronal image (B) axial image (C) sagittal image. A soft tissue mass is derived from the right nasal septum (yellow arrows in images A, B, and C). Bone destruction and sinus invasion are not confirmed. D. Nasal septum deviates to the left side (intact side).

Computed tomography revealed a soft tissue mass clinging to the right nasal septum without any signs of bone destruction and sinus invasion (Figures 2A-2C). Additionally, the nasal septum deviated to the left side (Figure 2D).

**Figure 3:**
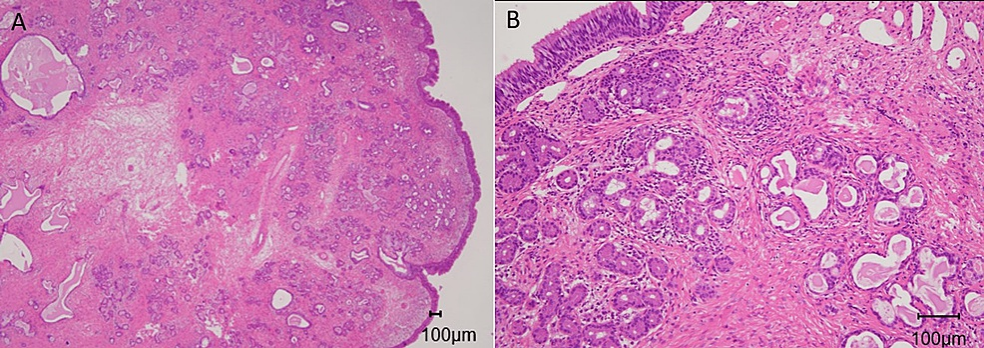
Histopathological findings A. The lesion is covered with respiratory epithelium, and the stroma is atrophied. (Hematoxylin and Eosin, X20) B. The stroma is filled with much fine collagen and moderate plasma cells and lymphocytes infiltrate while there are a few eosinophils. (Hematoxylin and Eosin, X100)

We excised the mass endoscopically along with its base, including the surrounding healthy mucosa of the right septum under local anesthesia (Figure 1B). We decided on the excision areas by distinguishing the tumor from the healthy mucosa endoscopically without pathological findings. There was little bleeding during the surgery, and we did not insert packing materials in the nostril. From its appearance, we expected the tumor to be not an ordinary inflammatory polyp. A pathological test (Hematoxylin & Eosin) revealed the atrophied stroma filled with fine collagen, and moderate plasma cells or lymphocytes infiltrate. There were a few eosinophils in the stroma. Respiratory epithelium covered the total tissue (Figures 3A, 3B).

Any signs of dysplasia or malignancy were not found. At the three-week visit, the lesion was replaced with healthy mucosa (Figure 1C).

## Discussion

We experienced the case of a patient with a septochoanal polyp on the concave side of the deviated nasal septum. The pathogenesis is still unknown, although the pathological findings suggest it is an inflammatory change. It is worth considering that the increased air stream on the concave side might have triggered the inflammation, forming a septochoanal polyp.

There have been only 13 cases of septochoanal polyp reported in the English literature. Four cases are men, five cases are women, and other cases are unknown [1-10]. No case of a child have yet been documented in the English article, while antrochoanal polyp is more common in men and children [11].

Although the pathogenesis is still unknown, allergy and mechanical pressure are thought to be the potential cause. Cook et al. reported that antrochoanal polyp was associated with allergic disease, including allergic rhinitis, bronchial asthma, and syndrome of aspirin sensitivity [11]. But the number of eosinophils in the pathological specimen of antrochoanal polyp is relatively low [10]. Furthermore, in the septochoanal polyp, no article shows abundant eosinophils in the polyp histologically, and only one patient had allergic rhinitis [3]. Our patient didn't have allergic diseases, and a histological examination revealed a few eosinophils in the stroma. Septochoanal polyp might not be associated with the allergy.

On the other hand, mechanical pressure might be related to the occurrence of septochoanal polyps. Most nasal polyps arise from the lateral walls of the nasal cavity, the anterior ethmoidal sinus mucosa, the contact areas of the uncinate process, and the middle turbinate [6]. These areas are exposed to more air turbulence than other structures in the nose. Irritants by the air stream can trigger inflammation [6]. In addition, nasal septum deviation changes the air stream in the nose. The airflow on the concave side becomes more substantial than that on the convex side [7]. Patients with antrochoanal polyps have larger maxillary sinus volume than ordinary people, and the polyps occur more frequently on the concave side of the deviated nasal septum [12]. Furthermore, the incidence of sinonasal papilloma is also higher on the concave side than on the convex side, which might be caused by the wall shear stress of the high-velocity airflow [13].

Our patient showed a septochoanal polyp occurring on the concave side of the deviated nasal septum. Unfortunately, we didn’t measure the airflow volume in our patient. But increased airflow in the right nasal cavity might have caused mucosa inflammation at the posterior nasal septum, leading to polyp formation. The relationships between the side of the septochoanal polyp and the direction of septal deviation have rarely been mentioned. Only one case in which a septochoanal polyp arose on the concave side has been documented in the English literature, while one is on the convex side [1,5]. The number of cases is too small to conclude, but the airflow effect is worth considering.

Pathological findings of choanal polyps are almost identical to those of an inflammatory polyp. They generally show inflammatory cell infiltration and edema in stroma while some reports point out that fewer eosinophils and submucous glands are confirmed in choanal polyps [14]. Histopathological examination is essential to differentiate from other tumors originating from nasal septa, such as epithelial adenomatoid hamartoma (REAH), chordoma, angiofibroma, paraganglioma, teratoma, and papilloma. Especially the most common origin of the REAH is the posterior nasal septum. We need to distinguish it from septochoanal polyp carefully. REAH tends to have a polypoid appearance and occurs in the paranasal sinuses, nasopharynx, and nasal cavities. Typical histological findings of the REAH are submucosal proliferative glands lined by ciliated respiratory epithelium originating from the surface respiratory epithelium [15]. The pathological findings in our patient revealed the atrophied stroma containing abundant fine collagen with moderate lymphocytes and plasma cell infiltration while a few eosinophils.

The standard treatment for choanal polyp is the surgical removal of the polyp. All 13 patients of septochoanal polyp underwent surgical resection. Additionally, there were no cases of recurrence in septochoanal polyp (follow-up period: 3-12 months), while the recurrence rate of the choanal polyp is reported to be 6 to 7.5% [1-10]. The prognosis of septochoanal polyps might be better because it is easy to excise the polyp completely due to anatomical position. Our patient didn't show any signs of recurrence at three weeks. We intend to continue follow-up for the time being.

## Conclusions

We encountered a 32-year-old man with a septochoanal polyp on the concave side of the deviated nasal septum. There are two possible causes for the development of such polyps, allergy and mechanical pressure. In the present case, increased airflow on the concave side might have caused inflammation of the mucosa at the posterior nasal septum, leading to polyp formation. We treated our patient by excising the polyp completely and our patient didn't show any signs of recurrence at three weeks follow-up.
